# Natural History of Diabetic Retinopathy Through Retrospective Analysis in Type 2 Diabetic Patients—An Exploratory Study

**DOI:** 10.3389/fpubh.2021.791378

**Published:** 2021-11-29

**Authors:** Mehak Gupta, Amarjeet Singh, Mona Duggal, Ramandeep Singh, Sanjay Bhadada, Poonam Khanna

**Affiliations:** ^1^School of Public Health, Postgraduate Institute of Medical Education and Research, Chandigarh, India; ^2^Advanced Eye Centre, Department of Ophthalmology, Postgraduate Institute of Medical Education and Research, Chandigarh, India; ^3^Department of Endocrinology, Postgraduate Institute of Medical Education and Research, Chandigarh, India

**Keywords:** diabetes—quality of life, diabetic retinopathy, proliferative and non-proliferative diabetic retinopathy, management, lifestyle

## Abstract

**Background and Aims:** Diabetic retinopathy is the most common diabetes-associated microvascular complication and is among the leading causes of vision loss or blindness in the adult population. The present study is a retrospective study that reported the natural history of diabetic retinopathy.

**Methods:** Retrospective medical records of 170 patients aged > 20 years with a confirmed complication of diabetic retinopathy were recruited into the present study. A questionnaire was also sent to each subject for gathering their experiences, and verification was done by the attending medical physicians. The questionnaire was answered by all recruited patients.

**Results:** The results showed that 23 (13.5%) subjects have a family history of diabetic retinopathy with 10 (5.9%) having mild NPDR, 63 (37.1%) with moderate NPDR, 60 (25.3%) have severe NPDR while 37 (21.8%) have PDR complications. The presence of co-morbidities was found in 139 (81.8%) subjects. Patients with PDR reported a significantly longer duration of diabetes mellitus with worse glycemic control.

**Conclusions:** The study revealed and concluded that adherence to the prescribed management regimen is important, for which patient education was the key which was lacking.

## Introduction

Diabetes mellitus (DM) is among the largest occurring non-communicable diseases worldwide associated with impairment in metabolism. With the increase in incidence and prevalence of DM, the associated micro-and macro-vascular complications, including diabetic retinopathy, diabetic nephropathy, and diabetic neuropathy along with cardio-vascular diseases have also shown an increased trend. Diabetic retinopathy (DR) is among the leading cause of vision loss in many countries (1). Among people aged between 20 and 79 years, it was found that the prevalence of DR was 35% (2). The longer the duration of DM, the higher the prevalence of the DR. There is still a scarcity of data related to incidence and progression of DR. Subjects with diabetes mellitus are 25 times more likely tend to become blind due to DR, which is why DR is among the priority diseases in the 2020 initiative for the global elimination of blindness.

Hyperglycemia in diabetes contributes to the damage of the tiny blood vessels of the retina that contribute to the progression of DR. DR leads to the leakage of fluid or hemorrhage (bleeding) that further distort the vision. In most of the advanced stages of the DR, blood vessels showed proliferation on the surface of the retina leading to cells loss and blindness. The present study aims to describe the natural history of diabetic retinopathy in the Indian population with T2DM and also to analyze the factors associated with the progression of DR.

## Materials and Methods

A cross-sectional facility-based study including retrospective history-based data collection on Diabetic retinopathy patients (>20 years of age) with type 2 diabetes was conducted at the Advanced Eye Centre PGIMER, Chandigarh. The data collection was carried out from May 13 to June 15, 2019, and analysis was completed in September 2019. The study population included all eligible DR patients (>20 years of age) attending the Diabetic retinopathy outpatient clinic in Advanced Eye Centre in PGIMER during the study period. Ethical clearance was taken from the intramural ethics committee of the Post Graduate Institute of Medical Education and Research (PGIMER), Chandigarh (India). Written informed consent of the patients was sought before the interviews. Participants were assured of confidentiality and were informed that they can withdraw from the process at any time during the study. The sample size was not separately calculated. The total enumeration technique was used based on the time available and as per the expected footfall of DR patients in the outpatient Retinopathy clinic in AEC, PGIMER. The average footfall for DR patients was 10 patients per OPD day. Approximately 240 patients were expected (24 working days), of these, 170 patients were recruited into the study. The rest either refused an interview, did not come under the inclusion criteria, or did not reach the room where the researcher was stationed. The patients who gave their consent were recruited for interviews in the study. Only patients who reported with type 2 diabetes, above the age of 20, years and diagnosed with DR, who were already on OPD follow-up visit (minimum 3 months of follow-up) in the Advance Eye Centre were included to take part in the study.

Patients were interviewed to elicit socio-demographic information, history of diabetes including onset history and family history of diabetes, degree of adherence to the treatment or management regime and co-morbidities, history of DR onset, the severity of the DR, family history of the DR, visual symptoms or impairment associated with DR, and other disease-related profiles. The patients' data were collected during the period between May 13, 2019 and June 15, 2019, with the permission of the advanced eye care center doctors and physicians. All the concerned doctors dealing with DR patients were requested to refer the diagnosed patients, after initial workup and prescription to the investigator. The recruited subjects were asked and informed for written consent. The patients who were vocal, willing, and forthcoming were subjected to in-depth interviews (IDI). Out of the total 170 interviews, IDI were conducted with 18 patients following the redundancy principle until the saturation point was reached, i.e., till no new information emerged.

Descriptive statistics were used to describe the socio-demographic profile. For the qualitative part, textual analysis of the verbatim responses from the respondents regarding the adherence problems faced was done through coding, indexing, and thematic extractions. The Chi-square test was used to calculate the significance between the degree of adherence to the management of the regime for diabetes mellitus and DR.

## Results

Table 1 shows the demographic characteristics of the study participants. The ages of the study participants ranged from 29 to 80 years, with a mean age of 57.2years (SD: 11.1). Of these 60 (35.3%) were women, and 110 (64.7%) were men. All told 7 (4.1%) were living alone while 81 (47.6%) lived in a joint family, 25 (14.7%) lived with only their spouse, 50 (29.4%) lived with their spouse and children, and 7 (4.1%) lived only with their kids. A total of 27 (15.9%) were illiterate, 23 (13.9%) completed school up to primary level, 69 (40.6%) were schooled to an intermediate level, and 51 (30%) were graduates and above. Regarding employment status: 56 (29.4%) were unemployed (which included housewives and people who could not work due to old age or any disability), 17 (10%) were laborers, 70 (41.2%) were either Government or private employees or had their own business (which included farmers), and 33 (19.4%) were retired and supported by a pension. Regarding income, 22 (12.9%) had a monthly family income below 10,000 INR and 40 (23.5%) had income more than 40,000 INR. In total 119 (70%) were urban residents. It was found that 102 (60%) were non-smokers and 82 (48.2%) did not drink alcohol. Regarding bodyweight, it was found that 11 (6.5%) were under-weight, 65 (38.2%) had normal BMI, 64 (37.6%) were over-weight and 30 (17.6%) were obese. Out of the total sample, 51 (30%) patients were referred from the endocrinology department of PGI.

**Table 1:** Demographic details of the respondents.

**Variables**		**Frequency (*N* = 170)**	**Percent (%)**
Age (years)	20–40	12	7.1
	40–60	94	55.3
	>60	64	37.6
Gender	Male	110	64.7
	Female	60	35.3
Marital status	Unmarried	4	2.4
	Married	139	81.8
	Divorced/separated	9	5.3
	Widow/widower	18	10.6
Living arrangement	Alone	7	4.1
	Joint family	81	47.6
	With spouse	25	14.7
	With spouse and kids	50	29.4
	With kids	7	4.1
Education	Illiterate	27	15.9
	Primary	23	13.5
	Matric	41	24.1
	Intermediate	28	16.5
	Graduate and above	51	30.0
Occupation	Unemployed/home duties	50	29.4
	Labor	17	10.0
	Private/govt/business	70	41.2
	Retired	33	19.4
Income (INR)	<10,000	22	12.9
	10,000–20,000	35	20.6
	20,000–40,000	73	42.9
	>40,000	40	23.5
Locality	Rural	51	30.0
	Urban	119	70.0
Smoking history	Non-smoker	102	60.0
	Current	41	24.1
	Past	27	15.9
Alcohol intake history	Non-alcoholic	82	48.2
	Current	54	31.8
	Past	34	20.0
BMI	Underweight	11	6.5
	Normal	65	38.2
	Overweight	64	37.6
	Obese	30	17.6
Referral To AEC	PGI (Endocrinology department)	51	30.0
	Outside PGI	119	70

Table 2 shows the history related to Diabetes of the respondent, which is the duration of Diabetes Mellitus (DM) diagnosis (in years), their then-current treatment regimen for the treatment of diabetes, and family history of diabetes. The average age for the development of type 2 DM was 45 years (SD 9.53). Table 3 shows the history related to Diabetic Retinopathy (DR) of the respondent which includes the duration of DR diagnosis (in years), family history of DR, severity of DR, presence of Macular edema, glycemic control during DR, and visual symptoms associated with DR. The average age for the development of any type of DR was 57.20 years (SD 11.07). Table 4 below shows that 76 (44.7%) patients had a history of cataracts and 139 (81%) patients had co-morbidities present along with DR. Table 5 demonstrates the availability of social support with the respondents, 77.1% of patients were accompanied by someone (1°, 2° or others) to the eye center, 81.2% had someone to help them in their activities of daily life, and 95.3% had someone to help them financially in times of need. Table 6 demonstrates that only 23 (13.5%) respondents were covered by any insurance while 81 (47.6%) respondents took reimbursement for the expenditure incurred for DR treatment. Table 7 shows the average age for the diagnosis of DM from birth and the average age for the diagnosis of DR from the birth of the respondents. Table 8 shows a natural history of DR in terms of the average time for the occurrence of different levels of severity of DR from the diagnosis of type 2 DM, i.e., a differential course. The mean duration of diabetes in mild NPDR, moderate NPDR, severe NPDR, and PDR diagnosis was 8, 8.1, 9, and 9.5 years, respectively, in our study.

**Table 2:** History related to diabetes.

**Variables**		**Frequency (*N* = 170)**	**Percent (*N*%)**
Duration of diabetes (years)	<5	18	10.6
	5–10	70	41.2
	10–20	16	36.5
	>20	20	11.8
Treatment for DM	OHA	84	49.4
	OHA + Insulin	86	50.6
Family history of diabetes	Yes	93	54.7

*OHA, Oral hypoglycemic agents*.

**Table 3:** History related to diabetic retinopathy.

**Variables**		**Frequency (*N* = 170)**	**Percent (%)**
Duration of DR (years)	<1	13	7.6
	1–3	66	38.8
	3–5	46	27.1
	>5	45	26.5
Family history of DR (*n* = 93)	Yes	23	13.5
Severity of DR	Mild NPDR	10	5.9
	Moderate NPDR	63	37.1
	Severe NPDR	60	25.3
	PDR	37	21.8
Presence of macular edema	Yes	116	68.2
Glycemic control	Good	94	55.3
	Poor	76	44.7
Visual symptoms	No symptom	30	17.6
	Blurry vision	48	28.2
	Watery eyes	11	6.5
	Floaters	47	27.6
	Itching	7	4.1
	Blood in eyes	27	15.9

**Table 4:** Other disease-related profile of the respondents.

**Variables**	**Frequency (*N* = 170)**	**Percent (%)**
H/O cataract	76	44.7
Presence of co-morbidities	139	81.8

**Table 5:** Social support available with the respondents.

**Variables**		**Frequency (*N* = 170)**	**Percent (%)**
Escorts with patient (relations)	1°	91	53.5
	2°	8	4.7
	Other	23	13.5
	1°and 2°	3	1.8
	1°and other	6	3.5
	None	39	22.9
Help in activities of daily life of patient (relations)	1°	98	57.6
	2°	3	1.8
	Other	2	1.2
	1° and 2°	30	17.6
	1° and other	5	2.9
	None	32	18.8
Financial help to patient (relations)	1°	122	71.8
	Other	8	4.7
	1° and 2°	9	5.3
	1° and other	23	13.5
	None	8	4.7

*1°realtions includes direct blood relations (parents, siblings, and children) and spouse. 2° relations includes close relatives. Other relations includes friends and distant relatives*.

**Table 6:** Financial security use by the respondent.

**Variables**	**Frequency (*N* = 170)**	**Percent (%)**
Insurance cover	23	13.5
Reimbursement taken	81	47.6

**Table 7:** Average age of the respondents at different stages of progression of DR.

**Average age at**	**Diagnosis of DM**	**Diagnosis of DR**
	45 ± 9.53 years	57.20 ± 11.07

**Table 8:** Average time for the occurrence of DR from DM.

**Severity Of DR**		**Average duration of DM (years)**	**Average duration of DR (years)**	**Average time for the occurrence of DR from DM**
NPDR	Mild (10)	9.8 ± 9.3	1.8 ± 2.6	8 ± 9.1
	Moderate (63)	11.17 ± 5.3	3.04 ± 2.7	8.12 ± 4.2
	Severe (60)	12.76 ± 5.7	3.75 ± 2.5	9.00 ± 4.6
PDR (37)		13.29 ± 5.5	3.77 ± 2.5	9.52 ± 4.1

*Number in parentheses represent the number of patients*.

Table 9 shows the degree of adherence to the prescribed management regime for the treatment of Type 2 DM. Only 2.9% of respondents adhered to their dietary modifications completely and just 1.8% of patients went for an eye examination for the screening of DR. Among the respondents, 52.4% completely adhered to drug intake, while only 21% adhered to exercise and glucose. Table 9A showed the chi-square “goodness of fit” test with a significant degree of adherence to the prescribed management regime for DR (*p* < 0.00001). Table 10 shows the degree of adherence to prescribed management regime for the treatment of DR. It shows that 41% of respondents adhere to dietary modifications completely, 94% were completely adherent to drug intake, and 39, 80, and 81% showed good compliance to exercise, glucose monitoring, and regular eye examination, respectively. Just 3% of patients went for an eye examination for the screening of DR. A total of 89% completely adhered to drug intake while only 35 and 36% adhered to exercise and glucose monitoring, respectively. Out of 152 patients prescribed for laser treatment, 98% adhered to it. Out of 119 patients prescribed for laser treatment, 71% showed good compliance while of 32 patients prescribed for surgery, only 59% showed adherence. It was found that 75% of patients maintained regular follow-up. Tables 10A,B showed the chi-square “goodness of fit” test with a significant degree of adherence to the prescribed management regime for DR (*p* < 0.00001). Table 11 shows the demographic profile of patients depending upon the severity of DR. This reflects the effect of different variables like age, gender, education, locality, history of smoking and alcohol intake, family history of DM & DR, presence of ME, history of cataracts, presence of co-morbidities on the severity of DR. Table 12 shows the degree of adherence the prescribed management regime according to the severity of DR. It was found that 32% patients with PDR poorly adhered to the diet, 80% of patients with mild NPDR showed good compliance to prescribed medications compared with only 40% of PDR patients. Again, 40% of patients with mild NPDR showed good compliance to regular glucose monitoring compared to 13% of PDR patients. Additionally, 92% of PDR patients showed poor adherence to the eye examinations. Another finding in our study is that patients referred to AEC from other departments within PGI are at milder stages of DR compared to patients referred from outside PGI, which is shown in Table 13. Similarly, Table 14 shows the ratio of patients with the presence of ME, referred to AEC from other departments within PGI was less compared to those referred from outside PGI.

**Table 9:** Degree of adherence to prescribed management regime for Type 2 DM.

**Prescribed management (*N*)**	**Degree of adherence**					
	**Good**		**Partial**		**Poor**	
	***n***	**(%)**	***N***	**%**	***n***	**%**
Dietary practices (170)	5	2.9	118	69.4	47	27.6
Exercise (170)	35	20.6	76	44.7	59	34.7
Drugs (170)	89	52.4	71	41.8	10	5.9
Glucose monitoring (170)	36	21.2	119	70.0	15	8.8
Eye examination (170)	3	1.8	31	18.2	136	80.0

*Number in parentheses represent the number of patients who were prescribed a particular regime*.

**Table 9A:** Chi-square test showing the degree of adherence to prescribed management regime for type 2 diabetes mellitus.

**Prescribed management**	**Degree of adherence**			
	**Good**	**Partial**	**Poor**	**Row totals**
Dietary practices	5 (33.60) [24.34]	118 (83.00) [14.76]	47 (53.40) [0.77]	170
Exercise	35 (33.60) [0.06]	76 (83.00) [0.59]	59 (53.40) [0.59]	170
Drugs	89 (33.60) [91.34]	71 (83.00) [1.73]	10 (53.40) [35.27]	170
Glucose monitoring	36 (33.60) [0.17]	119 (83.00) [15.61]	15 (53.40) [27.61]	170
Eye examination	3 (33.60) [27.87]	31 (83.00) [32.58]	136 (53.40) [127.77]	170
Column totals	168	415	267	850 (grand total)

*The chi-square statistic is 401.0703. The p-value is < 0.00001. The result is significant at p < 0.05*.

**Table 10:** Degree of adherence to prescribed management regime for DR.

**Prescribed management (*N*)**	**Degree of adherence**					
	**Good**		**Partial**		**Poor**	
	***n***	**(%)**	***n***	**%**	***N***	**%**
Dietary practices (170)	70	41	99	58	1	1
Exercise (170)	66	39	81	48	23	13
Drugs (170)	159	94	11	6	1	0.6
Glucose monitoring (170)	136	80	34	20	1	0.6
Eye examination (170)	137	81	31	18	2	1
Laser (152)	149	98	3	2	1	0.65
Anti-VEGF (119)	85	71	18	15	16	14
Surgery (39)	23	59	1	2.56	16	41
Regular follow-up (170)	127	75	42	25	1	1

*Number in parentheses represent the number of patients who were prescribed a particular regime*.

**Table 10A:** Chi-square test showing the degree of adherence to prescribed management regime for DR.

**Degree of adherence**				
**Prescribed management**	**Good**	**Partial**	**Poor**	**Row totals**
Dietary practices	70 (113.33) [16.57]	99 (51.08) [44.96]	1 (5.59) [3.77]	170
Exercise	66 (113.33) [19.77]	81 (51.08) [17.53]	23 (5.59) [54.27]	170
Drugs	159 (114.00) [17.76]	11 (51.38) [31.74]	1 (5.62) [3.80]	171
Glucose monitoring	136 (114.00) [4.25]	34 (51.38) [5.88]	1 (5.62) [3.80]	171
Eye examination	137 (113.33) [4.94]	31 (51.08) [7.89]	2 (5.59) [2.30]	170
Column totals	568	256	28	**852 (grand total)**

*The chi-square statistic is 239.2154. The p-value is < 0.00001. The result is significant at p < 0.05*.

**Table 10B:** Chi-square test showing the degree of adherence to prescribed management regime for DR.

**Degree of adherence**				
**Prescribed management**	**Good**	**Partial**	**Poor**	**Row totals**
Laser	149 (121.89) [6.03]	3 (20.32) [14.76]	1 (10.79) [8.89]	153
Anti-VEGF	85 (94.80) [1.01]	18 (15.80) [0.31]	16 (8.39) [6.89]	119
Surgery	23 (31.87) [2.47]	1 (5.31) [3.50]	16 (2.82) [61.55]	40
Regular follow-up	127 (135.44) [0.53]	42 (22.57) [16.72]	1 (11.99) [10.08]	170
Column totals	384	64	34	**482 (Grand Total)**

*The chi-square statistic is 132.7225. The p-value is < 0.00001. The result is significant at p < 0.05*.

**Table 11:** Socio-demographic profile of patients depending upon the severity of DR.

**Demographic details**		**NPDR**			**PDR% (*n* = 37)**
		**Mild % (*n* = 10)**	**Moderate % (*n* = 63)**	**Severe% (*n* = 60)**	
Average age (years)		40.9 ± 10.1	55 ± 9.97	59.5 ± 9.6	60.7 ± 11.25
Age (years)	20–40	60	6	2	3
	40–60	40	70	52	51
	>60	0	32	46	46
Gender	Male	60	60	65	73
	Female	40	40	35	27
Education	Illiterate	0	13	20	19
	Primary	10	8	17	19
	Matric	10	22	23	32
	Intermediate	20	16	18	16
	Graduate and above	60	41	23	14
Locality	Rural	30	19	32	46
	Urban	70	81	68	54
Smoking history	Non-smoker	10	60	53	62
	Past	90	13	17	22
Alcohol intake history	Non alcoholic	60	19	53	43
	Past	10	49	17	30
BMI	Underweight	0	3	10	43
	Normal	50	36	43	30
	Overweight	50	37	33	8
	Obese	0	24	14	19
Referral to AEC	PGI (Endocrinology department)	80	38	27	92
	Outside PGI	20	62	73	8
Duration of diabetes (years)	≥10	80	62	47	34
	<10	20	37	53	66
Treatment for DM	OHA	100	70	32	3
	OHA + Insulin	0	30	68	97
Family history of diabetes	Yes	8	64	50	40
Duration of DR (years)	≥3	80	54	36	40
	<3	20	46	64	60
Family history of DR (*n* = 93)	Yes	50	10	13	13
Presence of macular edema	0	0	46	87	95
Visual symptoms	No symptom	60	29	8	3
	Blurry vision	40	30	28	21
	Watery eyes	0	9	6	3
	Floaters	0	24	25	46
	Itching	0	6	5	0
	Blood in eyes	0	2	26	27
H/O cataract		0	36	51	60
Presence of co-morbidities		40	76	92	84

**Table 12:** Degree of adherence to prescribed management regime according to severity of DR.

**Adherence to prescribed management**		**NPDR**			**PDR% (*n* = 37)**
		**Mild % (*n* = 10)**	**Moderate % (*n* = 63)**	**Severe% (*n* = 60)**	
Dietary practices	Good	0	8	0	0
	Partial	90	67	70	68
	Poor	10	25	30	32
Exercise	Good	30	27	12	22
	Partial	40	41	50	43
	Poor	30	32	38	35
Drug intake	Good	80	62	45	40
	Partial	20	36	48	46
	Poor	0	2	7	14
Glucose monitoring	Good	40	29	15	13
	Partial	60	68	77	70
	Poor	0	3	8	22
Eye examination	Good	0	3	2	0
	Partial	30	24	17	8
	Poor	70	73	81	92

**Table 13:** Association between severity of DR and referral to AEC.

		**Severity of DR**				**Total**
		**NPDR**			**PDR**	
		**Mild**	**Moderate**	**Severe**		
Referral to AEC	Outside PGI	2	39	44	34	119
	Within PGI	8	24	16	3	51
Total		10	63	60	37	170

**Table 14:** Association between the presence of Macular edema and referral to AEC.

		**Presence of macular edema**		**Total**
		**YES**	**NO**	
Referral to AEC	Outside PGI	97	22	119
	Within PGI	19	32	51
Total		116	54	170

## Discussion

Diabetic Mellitus is a chronic disease associated with abnormally high levels of glucose in the blood. It occurs due to either inadequate production of insulin (which is made by the pancreas and lowers blood glucose), or due to inadequate sensitivity of cells to the action of insulin. In type 2 diabetes, there is generally enough insulin but the cells upon which it should act are not normally sensitive to its action. It is also called adult-onset diabetes. Nine out of ten people with diabetes have type 2 diabetes (3). It occurs most often in people who are over 40 years old however, it can occur even in childhood if there are risk factors present. In our study the average age for the development of type 2 diabetes was 45 years (3).

DM is a rapidly growing health issue with a total of 69 million type 2 diabetic patients in India, which is expected to rise to 98 million by 2030 (4). DM is a lifelong illness that requires continuing medical care. Most hospitals focus on patient self-management education strategies to prevent acute complications and reduce the risk of long-term complications. DM is associated with many microvascular (retinopathy, neuropathy, and nephropathy) and macrovascular (stroke, ischemic heart disease, and peripheral artery disease) complications. One of the most common microvascular complications of DM is DR, which is one of the leading causes of vision loss. Two-thirds of all type 2 DM patients develop DR (5). Hence it is important to analyze the problem so that strategies may be established to reduce its growing burden. There are different stages of DR depending upon the severity of the disease, these are mild NPDR, moderate NPDR, severe NPDR, and PDR (6). The severity of these stages also depends upon the presence of Macular edema (6). Mild and moderate NPDR are the less severe stages and can be managed by maintaining good glycemic control. These are usually not accompanied by ME and do not require any surgical treatment. The symptoms for DR mostly appear in the later stages like severe NPDR or PDR and are more extenuated in presence of ME (7). The symptoms mainly include vision loss, floaters, blood in the eyes, watery eyes, and itching; all of which require treatment (Laser therapy, Anti-VEGF injections, or surgery).

Some of the risk factors for the development of DR are the duration of diabetes, glycemic control, family history, presence of co-morbidities, smoking history, and history of alcohol intake (8). According to our study, it took an average of 8.7 years for the development of any type of DR from type 2 DM. The mean duration of diabetes in mild NPDR, moderate NPDR, severe NPDR, and PDR diagnoses was 8, 8.1, 9, and 9.5 years, respectively, in our study. This implies that over time gradual damage occurs in the retina leading to various levels of severity in DR.

The management regime for type 2 DM patients includes lifestyle modification and intake of hypoglycemic drugs (9). Lifestyle modifications include dietary modification, regular exercise, cessation of smoking and alcohol, and timely glucose monitoring, with an annual full body checkup including eye checkup (10). DR is usually a result of uncontrolled diabetes. Uncontrolled diabetes is mainly because of non/poor adherence to the treatment prescribed. By and large, most people find it difficult to adhere to such a rigorous regime. This is because adherence requires a significant change in the routine of their life.

Our results indicate that the adherence profile changed significantly in the patients after the development of DR. The results showed that only 3% of respondents adhered to dietary practices before DR, which rose to 21% after DR. The most common reason given by the respondents for non-adherence was a temptation to eat certain foods. Other common reasons included peer pressure during family gatherings or functions. The influence of habits of their family members or friends who are diabetic also affects adherence. The degree of adherence to exercise was 21%, which increased to 39%. The presence of comorbidities and giving less importance to exercise were the main reason for non-adherence to exercise. Reduction in visual acuity, lack of time, and lack of a conducive environment were some other reasons. A total of 52% of the respondents adhered to the prescribed medication. Only 21% were adherent to glucose monitoring before DR. The reason given for non-adherence was forgetfulness, not feeling the need, lack of family support, and carelessness. The adherence rate increased to 92% for medication use and 80% for glucose monitoring after diagnosis of DR. Education and family support play a crucial role in motivating people to make necessary lifestyle changes in managing a chronic illness (11).

Just 3% of patients adhered to eye examinations before a diagnosis of DR, which rose to 83% after it. The main reason for such low adherence rates was a lack of awareness of diabetic eye complications. The other important reason was the non-appearance of any visual symptom other than weak eyesight in the early stages of DR. In most of the patients it is only in the later stages of DR that some significant symptoms appear like floaters and blood in the eyes which make the patient seek medical care. There is a direct link between the severity of disease and the inclination to seek care.

According to our results, we can classify determinants of the differential course of DR into (12) Modifiable factors, and, (13) Non-modifiable factors. Modifiable factors can further be divided into (a) Patient-related, and (b) Provider-related. Age and genetics are non-modifiable factors because these cannot be altered. Obesity, the presence of co-morbidities, carelessness, smoking, or alcohol addiction can be classified under modifiable patient-related factors as these can be controlled by the patients. These can be modeled through behavior change. Lack of knowledge as revealed by some patients —*humepta hi nahithaki sugar se ankhebhikharabhotihai*|| (we did not know that diabetes could affect eyes), “*sabse ache sugar ke doctor kodikhate the hum usnekabinaikahakiankhe test karlenichahiye”*(We used to consult the best endocrinologist, he never told us to go for an eye checkup), lack of focus on health education and motivation, and lack of time given by provider can be put under modifiable provider side factors. This indicates that the health care delivery system failed on its part. Doctors/nurses/caregivers are expected to educate and tell people about their illness and its related complications. They did not do it effectively.

The significant increase in the adherence pattern of the patients before and after DR may be attributable to the importance of one's eyes in one's life (14, 15). Eyes are not just for sight, they are the portal through which the brain can tell us about the world around us, through which we can learn new things, and make wonderful memories. Seeing the surrounding world is undoubtedly a wonderful experience. Throughout history, deprivation of eyesight has been perceived as a most severe form of punishment, often second only to loss of life. It is of utmost importance for survival. Most people will make definite efforts to retain their eyesight. Our study also showed a significant increase in adherence after the diagnosis of DR. A 45 year man stated that —*ankhokebinainsankyahai*|| (What is a man without eyes). A 50-year-old woman commented that “*Andhi ho gayi to doosrokemohtaj ho jayugi*|| (If I go blind I will be dependent on others). The threat of losing an invaluable sense organ is one trigger of behavioral change. “*Ankhena hone se achahai hum mar jaye*|| (It‘s better to die than to go blind)” was one comment made by a 42-year-old respondent suffering from PDR.

During informal interactions with the patients, there was a wide range of reasons given by them for the etiology of diabetes such as excessive consumption of sweet foods or sugary things, lack of sleep, taking medication for other illnesses, stress, previous long illness, fate, and many others. There were a few patients (39%) who were aware of the possible etiological factors (family history, sedentary lifestyle, age, psychological factors) and about symptoms related to type 2 diabetes. The knowledge that these patients acquired was mostly from their friends or the people around them. Mainly, it was only these patients who had observed that there was something unusual in their health and consulted the doctor about it. In other patients with low knowledge about diabetes, the detection was either incidental “*Friend kesathgayatha checkup karneapnabhikaralia”* (I went for my friend‘s checkup. Got mine also done) as one 58-year-old man told. “*Heart attack huatha tab ptachala sugar hai”* (Diabetes was detected in tests during heart-attack) remarked another 68-year-old man, or diagnosed when the serious symptoms appeared like delayed wound healing, weakness, or some infection. “*Pair me kantachubathaaurzakhambadhraha ha”* (Got hurt by a thorn in the foot and the wound enlarged instead of getting healed) narrated one 62-year-old man.

On asking about DR most of the patients had not heard about the terminology DR but they were aware that sugar had affected their eyes. During the interview, most respondents knew that uncontrolled diabetes was the cause for the genesis of DR. They admitted that if they had known this earlier they could have made serious amendments in their lifestyle to undo it. “*Agr Hume pheleptahotaki sugar se ankhekharabhotihai to hum phele hi dhyanrakhte*.” Some patients blamed fate for their condition. “*kismat me likhahoga”* (This might be in my destiny) and some blamed their doctors for not giving them adequate knowledge about DR. “*doctor kobatanachahiye the”* (Doctor should have told us). The most common answer given by the majority of the respondents for the etiology of DR was some kind of stress. Unawareness was one of the main factors which prevented people from going for an eye checkup.

Our study revealed that adherence to the prescribed regimen is important, for which patients' education is the key which was lacking. The knowledge about diabetes and DR was very poor in the patients. Our results indicate that all said and done even in the twenty-first century where all the buzz is all about NCDs and their prevention, people have low levels of knowledge about diabetes and its complications.

It is necessary to address the gaps between knowledge and awareness levels amongst patients. Improvement of knowledge and awareness is needed for patients. This will help patients to optimize their lifestyles and improve their medication habits and delay the onset of long-term complications. This becomes important on the part of healthcare providers to give early diabetic education regarding the causes, management, and preventive measures of diabetic complications. Focus on health education and preventive measures such as adjusting to lifestyle modifications and adhering to proper treatment will enhance the level of knowledge and confidence among diabetic patients to live a healthy life.

A DM patient may not perceive themself as susceptible to DR due to ignorance or a lack of adequate information provided by the concerned doctors. Consequently, they may be casual regarding how rigorously they adhere to the prescribed regimen. But once the DR sets in, the involvement of eyes, the crucial sense organs, their Perceived Seriousness is quite high, warranting a much better adherence to the prescribed regimen. The health belief model can explain these concepts.

## Conclusions

The present study concluded that the DR complication is associated with the long-term history of diabetes mellitus that further presents into mild, moderate, severe, and proliferative diabetic retinopathy with the progression of years for diabetes mellitus. Age, genetics, presence or absence of co-morbidity along with lifestyle modifications and adherence to the management of DR or diabetes mellitus contribute heavily in the management or treatment of DR patients. The adherence profile significantly contributes to the development of the DR. Education and awareness of diabetes and its associated complications also play important role in the progression of DR.

## Data Availability Statement

The raw data supporting the conclusions of this article will be made available by the authors, without undue reservation.

## Ethics Statement

The studies involving human participants were reviewed and approved by Ethics Committee PGIMER. The patients/participants provided their written informed consent to participate in this study.

## Author Contributions

MG was involved in manuscript writing, conceptualization, and data analysis. SB, AS, RS, MD, and PK supervised and reviewed the manuscript. All authors contributed to the article and approved the submitted version.

## Conflict of Interest

The authors declare that the research was conducted in the absence of any commercial or financial relationships that could be construed as a potential conflict of interest.

## Publisher's Note

All claims expressed in this article are solely those of the authors and do not necessarily represent those of their affiliated organizations, or those of the publisher, the editors and the reviewers. Any product that may be evaluated in this article, or claim that may be made by its manufacturer, is not guaranteed or endorsed by the publisher.
